# The cost of influenza-associated hospitalizations and outpatient visits in Kenya

**DOI:** 10.1186/s12889-019-6773-6

**Published:** 2019-05-10

**Authors:** Gideon O. Emukule, Linus K. Ndegwa, Michael L. Washington, John W. Paget, Jazmin Duque, Sandra S. Chaves, Nancy A. Otieno, Kabura Wamburu, Irene W. Ndigirigi, Philip M. Muthoka, Koos van der Velden, Joshua A. Mott

**Affiliations:** 1 Centers for Disease Control and Prevention - Kenya Country Office, KEMRI Headquarters, Mbagathi Rd, Off Mbagathi Way, Village Market, P. O Box 606, Nairobi, 00621 Kenya; 2 National Center for Emerging and Zoonotic Diseases, US Centers for Disease Control and Prevention, Atlanta, GA USA; 3 Netherlands Institute for Health Services research (NIVEL), Utrecht, The Netherlands; 4 Influenza Division, National Center for Immunization and Respiratory Diseases, US Centers for Disease Control and Prevention, Atlanta, GA USA; 5 Kenya Medical Research Institute, Kisumu, Nairobi Kenya; 6 Ministry of Health, Government of Kenya, Nyeri, Nairobi Kenya; 7 Radboud University Medical Center, Department of Primary and Community care, Nijmegen, The Netherlands; 8 US Public Health Service, Rockville, MD USA

**Keywords:** Economic burden, Cost, Cost-per-episode, Hospitalization, Influenza, Kenya, Outpatient

## Abstract

**Background:**

We estimated the cost-per-episode and the annual economic burden associated with influenza in Kenya.

**Methods:**

From July 2013–August 2014, we recruited patients with severe acute respiratory illness (SARI) or influenza-like illness (ILI) associated with laboratory-confirmed influenza from 5 health facilities. A structured questionnaire was used to collect direct costs (medications, laboratory investigations, hospital bed fees, hospital management costs, transportation) and indirect costs (productivity losses) associated with an episode of influenza. We used published incidence of laboratory-confirmed influenza associated with SARI and ILI, and the national population census data from 2014, to estimate the annual national number of influenza-associated hospitalizations and outpatient visits and calculated the annual economic burden by multiplying cases by the mean cost.

**Results:**

We enrolled 275 patients (105 inpatients and 170 outpatients). The mean cost-per-episode of influenza was US$117.86 (standard deviation [SD], 88.04) among inpatients; US$114.25 (SD, 90.03) for children < 5 years, and US$137.45 (SD, 76.24) for persons aged ≥5 years. Among outpatients, the mean cost-per-episode of influenza was US$19.82 (SD, 27.29); US$21.49 (SD, 31.42) for children < 5 years, and US$16.79 (SD, 17.30) for persons aged ≥5 years. National annual influenza-associated cost estimates ranged from US$2.96–5.37 million for inpatients and US$5.96–26.35 million for outpatients.

**Conclusions:**

Our findings highlight influenza as causing substantial economic burden in Kenya. Further studies may be warranted to assess the potential benefit of targeted influenza vaccination strategies.,

**Electronic supplementary material:**

The online version of this article (10.1186/s12889-019-6773-6) contains supplementary material, which is available to authorized users.

## Background

In Kenya, influenza virus circulates year-round and is an important contributor to the acute respiratory illness associated burden, disproportionately affecting children aged < 5 years [1, 2]. Despite the documented burden of influenza disease in Kenya, a national influenza vaccination program is yet to be implemented. Other than the health impact caused by influenza virus infection itself, influenza illness has been shown to exert a considerable economic burden, although most of the data available come from temperate and resource rich countries [3–6]. Understanding the costs of influenza-associated illness in Kenya is critical to allow health authorities and policy makers to develop practical plans for vaccine recommendations.

We estimated the cost-per-episode, from a societal perspective, of laboratory-confirmed influenza-associated illness in Kenya using data collected from interviews with case-patients or their care-takers, and abstracted from medical records. Additionally, we estimated the annual economic burden of influenza-associated illness in Kenya by applying the estimated costs to the annual national morbidity burden using previously published data on burden of influenza-associated disease.

## Methods

### Study sites and population

**Fig. 1:**
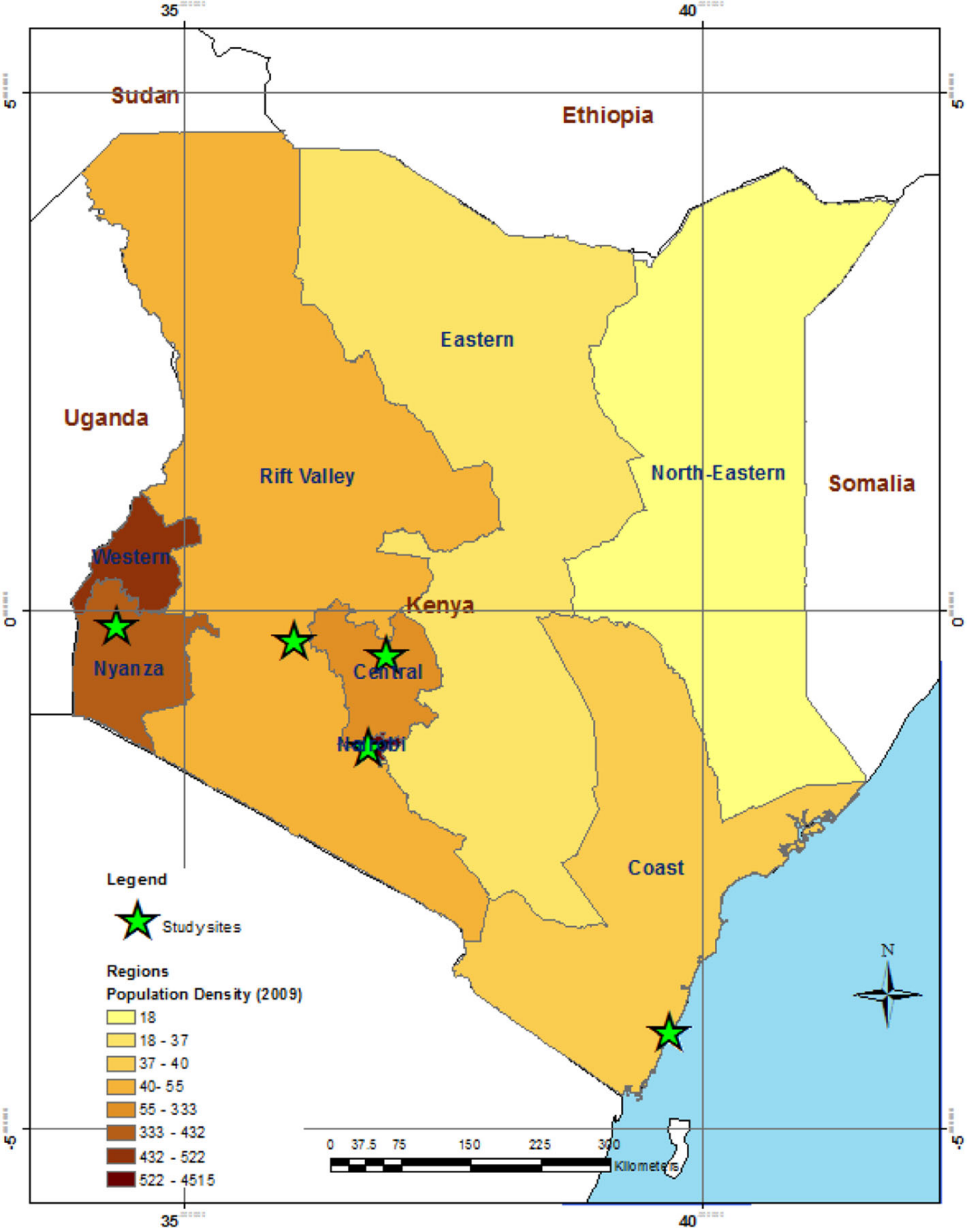
Map of Kenya showing the location of the study sites. Figure is of the authors’ own creation

From July 2013 through August 2014, we prospectively enrolled patients from four hospitals [Mombasa County Referral Hospital (CRH), Nakuru CRH, Nyeri CRH, and St. Elizabeth Mission Hospital in Lwak] and one outpatient facility (Tabitha clinic in Kibera) (Fig. 1). Mombasa CRH, Nakuru CRH and Nyeri CRH are public health facilities. Kibera clinic is located in an urban informal settlement in Nairobi and is operated by Carolina for Kibera [7]. St. Elizabeth Mission hospital is a rural site in western Kenya operated by the Franciscan Sisters of St. Anna [7]. The study sites were purposely selected for their diversity and their representation of populations in multiple geographical locations (Fig. 1).

Patients were enrolled if they met the case definitions for severe acute respiratory illness (SARI) or influenza-like illness (ILI) and tested positive for influenza A and/or B using the Becton Dickson (BD) Veritor™ rapid diagnostic test (RDT) in nasal swabs collected at interview [8, 9]. SARI was defined as hospitalization with an acute respiratory infection within the last ten days with a history of fever or measured temperature ≥ 38 C°, and cough. ILI was defined as an acute respiratory infection within the last seven days with a measured temperature ≥ 38 C° and cough.

### Confirmatory testing for influenza

Nasopharyngeal (NP) and oropharyngeal (OP) swabs were collected from all consenting and enrolled patients. The NP/OP swabs were combined into a single viral transport media, and tested by real-time reverse transcription polymerase chain reaction (rtRT-PCR) for influenza A and B virus at the Kenya Medical Research Institute (KEMRI) and U. S Centers for Disease Control and Prevention (CDC) laboratory in Nairobi [10]. Data from patients whose NP/OP specimens were confirmed to be positive for influenza virus by rtRT-PCR were used in the final data analysis.

### Data collection

Patients who were aged ≥18 years were interviewed directly by trained surveillance officers using a structured questionnaire. For those who were aged < 18 year, their care-takers were interviewed. Enrolled study participants were subsequently followed-up using telephone interviews to determine additional costs incurred over a period of 14 days from the date of testing for influenza. This period was chosen because most uncomplicated influenza infections resolve within a period of two weeks [11]. To minimize the possibility of recall bias, the follow-up telephone interviews were conducted on a weekly basis. The first interview was conducted on the 8th day (to cover the preceding 7 days); and the last on the 15th day (to cover the other 7 days). Data on clinical management of the patients were abstracted from the medical records of the case-patients (i.e., patient files and charge sheets). As study participants at the two population-based study sites receive free medical care provided by KEMRI and CDC, we used costs chargeable to non-study participants and costs of purchase provided by the study administrative staff at Lwak and Kibera, respectively.

### Direct and indirect cost components

The direct cost components included facility-based medical-cost items (i.e. medications, laboratory investigations and other routine diagnostics, hospital bed fees, and hospital management costs) (Additional file 2), and travel costs by the case-patients and/or their household members. Facility-based medical-cost items were obtained from the hospital bill charge sheets for inpatients, price catalogue charts, and receipts issued to the outpatients (Additional file 2 and Additional file 1). Other direct costs included costs incurred for seeking care prior and after discharge from the hospital or outpatient visit (e.g. over the counter prescriptions). Other than children < 5 years whose medical costs were paid for by the government in public health facilities, all costs (excluding consultation fees) were paid for out-of-pocket by older patients [12]. The costs of testing for influenza were not included as they are not routinely ordered by clinicians independently from the ongoing surveillance.

The indirect cost component was the productivity losses (days of work lost) at the household level by the case-patients themselves, and/or any of their household members (Additional file 1). Data on days of work lost were only considered for those who were engaged in formal or informal income generating employment who would otherwise not be financially compensated for the lost workdays.

### Data analyses

#### Descriptive analyses and tests of associations

Data on patient characteristics were described using proportions. Tests of association were performed using chi-square tests for categorical variables. For continuous variables, data were described using means, standard deviations (SD), medians, and interquartile ranges (IQR). Comparisons of means were done using the independent t-test or one-way analysis of variance, while medians were compared using Wilcoxon rank sum test or Kruskal-Wallis test as appropriate. Data analyses were performed using Stata version 13.0 (StataCorp. 2013. Stata Statistical Software: Release 13. College Station, TX: StataCorp LP).

#### Cost-per-episode of influenza-associated illness

The cost-per-episode of influenza-associated illness was estimated as the sum of the facility-based medical costs, household transportation costs, the costs of seeking health care prior to the current visit, and the household lost productivity cost (Additional file 1) [6, 13]. The routine service delivery cost at the facility – which included buildings and equipment maintenance, transport, electricity, water, fuel, communication, stationery, and wages for support staff – was estimated for each patient using health facility administration data collected over the financial year 2014 (Additional file 1). We also explored the option of using the WHO-Choice estimates for routine healthcare service costs for hospitalized patients (cost per bed day) and outpatients (cost per outpatient visit) [14]. We found minimal differences compared to when we used actual data and subsequently opted to report costs calculated using the actual routine healthcare service cost data collected from the study sites (Additional file 1). Definitions and data sources of the cost components are provided in Additional file 2.

#### National economic burden of influenza-associated illness

To estimate the annual economic burden of influenza-associated illness, we used the published national annual incidence (between 2007 and 2013) of influenza-associated hospitalizations and outpatient visits for children < 5 years and persons ≥5 years [15]. We carried out a sensitivity analysis for the best- and worst-case scenario assuming a low and high incidence of influenza-associated illness respectively [15]. We applied the incidence rates to the population size in 2014, projecting an annual growth rate of 2.7% from the 2009 national census, to estimate the annual number of hospitalizations and outpatient visits associated with influenza illness [16, 17]. We used bootstrap samples – with 1000 replications of the same size as the original dataset and sampled with replacement – to estimate the mean costs which were then applied to the hospitalizations and outpatient visits to estimate overall costs. All costs reported in our analysis are in United States (U.S.) Dollars (1 US$ = 90 Kenya Shillings in 2014).

## Ethical considerations

The KEMRI Ethical Review Committee (KEMRI SSC-2492) and Institutional Review Board of U. S CDC (CDC IRB # 6539) approved this study. Written informed consent was obtained from all participants or caretakers/guardians of all minors prior to enrolment in the study and sample collection.

## Results

### Descriptive analyses

**Fig. 2:**
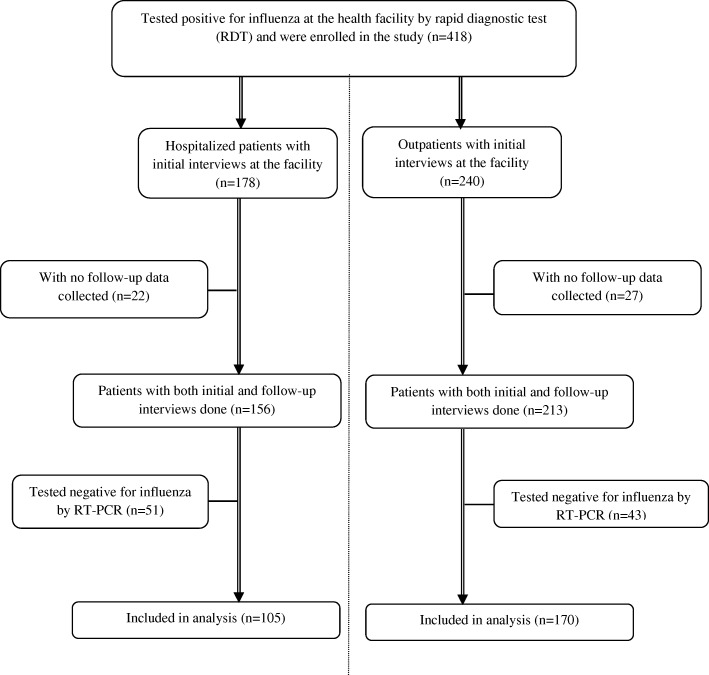
Data flow diagram

From July 2013 through August 2014, a total of 418 patients were initially recruited in the study. After excluding patients who tested negative for influenza by rtRT-PCR and those without follow up data, a total of 275 case-patients were included in the final analysis (Fig. 2 and Table 1). Among these were 105 inpatients (< 5 years = 88; ≥5 years = 17), and 170 outpatients (< 5 years = 112; ≥5 years = 58). Among the inpatients, 90/105 (85.7%) tested positive for influenza A only [influenza A(H1N1) pdm09 = 43, A(H3N2) =30, not subtyped = 17], 13/105 (12.4%) were influenza B only cases, and 2/105 (1.9%) tested positive for both influenza A and B. Of the outpatients included in the analysis, 149/170 (87.6%) were influenza A only cases [influenza A(H1N1) pdm09 = 67, A(H3N2) =50, not subtyped = 32], 20/170 (11.8%) were influenza B only cases, and 1/170 (0.6%) tested positive for both influenza A and B.

**Table 1:** General characteristics of study patients with influenza-associated illnesses in Kenya, July 2013–August 2014

Variable	All data (*n* = 275)	Inpatients			Outpatients		
		< 5 years (*n* = 88)	≥5 years (*n* = 17)	Total (*n* = 105)	< 5 years (*n* = 112)	≥5 years (*n* = 58)	Total (*n* = 170)
Hospital, n(%)							
Coast PGH	38 (13.8)	25 (28.4)	0 (0.0)	25 (23.8)	13 (11.6)	0 (0.0)	13 (7.7)
Nyeri PGH	35 (12.7)	28 (31.8)	5 (29.4)	33 (31.4)	2 (1.8)	0 (0.0)	2 (1.2)
Nakuru PGH	98 (35.6)	35 (39.8)	12 (70.6)	47 (44.8)	45 (40.2)	6 (10.3)	51 (30.0)
Tabitha Clinic (Kibera)	89 (32.4)	0 (0.0)	0 (0.0)	0 (0.0)	48 (42.9)	41 (70.7)	89 (52.4)
St. Elizabeth Hosp. (Lwak)	15 (5.5)	0 (0.0)	0 (0.0)	0 (0.0)	4 (3.6)	11 (19.0)	15 (8.8)
Sex of patient (Male), n(%)	135 (49.1)	50 (56.8)	7 (41.2)	57 (54.3)	58 (51.8)	20 (34.5)	78 (45.9)
Age of patient (< 5 years), n(%)	200 (72.7)	88 (100.0)	N/A	88 (83.81)	112 (100.0)	N/A	112 (65.9)
Relationship of respondent to patient, n(%)							
Self	31 (11.3)	0 (0.0)	8 (47.1)	9 (8.6)	0 (0.0)	22 (37.9)	22 (12.9)
Mother	222 (80.7)	81 (92.0)	8 (47.1)	88 (83.8)	105 (93.8)	29 (50.0)	134 (78.8)
Father	13 (4.7)	4 (4.6)	0 (0.0)	4 (3.8)	6 (5.4)	3 (5.2)	9 (5.3)
Other	9 (3.3)	3 (3.4)	1 (5.9)	4 (3.8)	1 (0.9)	4 (6.9)	5 (2.9)
Sought care prior to this hospitalization/outpatient visit, n(%)	107 (38.9)	58 (65.9)	10 (58.8)	68 (64.8)	34 (30.4)	5 (8.6)	39 (22.9)
Purchased any medications prior to hospital/clinic visit, n(%)	77/107 (72.0)	42/58 (72.4)	7/10 (70.0)	49/68 (72.1)	24/34 (70.6)	4/5 (80.0)	28/39 (71.8)
Sought care after discharge from the hospital/clinic, n(%)	26 (9.5)	8 (9.1)	3 (17.7)	11 (10.5)	11 (9.8)	4 (6.9)	15 (8.8)
Household member(s) missed work due to illness, n(%)	177 (64.4)	72 (81.8)	17 (100.0)	89 (84.8)	54 (48.2)	34 (58.6)	88 (51.8)
Total number of workdays lost in a household, median(IQR)	4 (3–8)	7 (4–10)	6 (3–13)	6 (4–10)	3 (2–6)	3 (2–5)	3 (2–5)
Average number of household workdays lost due to illness, median (IQR)^a^	4 (2–7)	5 (3–9)	6 (3–12)	5 (3–9)	3 (2–5)	3 (2–4)	3 (2–5)
Household member(s) missed school due to illness, n(%)	90 (32.7)	17 (19.3)	6 (35.3)	23 (21.9)	35 (31.3)	32 (55.2)	67 (39.4)
Total number of school days missed, median(IQR)	4 (3–6)	2 (1–6)	5 (4–9)	3 (1–6)	3 (2–6)	4 (3–7)	4 (3–6)
Paid for child care in the course of sickness, n(%)	6 (2.2)	3 (3.4)	0 (0.0)	3 (2.9)	1 (0.9)	2 (3.5)	3 (1.8)
Paid for child care in the course of sickness, n(%)	6 (2.2)	3 (3.4)	0 (0.0)	3 (2.9)	1 (0.9)	2 (3.5)	3 (1.8)

*NA* Not applicable; ^a^Calculated as the total number of workdays lost by all household members who reported to be engaged in an income activity divided by the number of persons who reported to be engaged in an income activity

The majority (73%) of the case-patients were children < 5 years. Among persons ≥5 years, median age was 11 years (interquartile range [IQR], 7–30); only 7 (9%) were aged ≥40 years. Overall 135 (49%) were males (Table 1). The median length of hospitalization was 4 days (IQR, 3–6); 5 days (IQR, 3–7) among children < 5 years compared to 4 days (IQR, 3–4) among older patients (*p* = 0.050). The average monthly household income was US$ 225.83 (20,325 Kenya shillings).

Forty six percent of the case-patients aged < 5 years were taken to care or had drugs bought for them over the counter prior to enrollment compared to 20% for persons aged ≥5 years (*p* < 0.001). The median (IQR) number of workday opportunity losses was 2 days (0–6); 5 days (2–9) among inpatients vs. 1 day (0–3) among outpatients (*p* < 0.001). The median (IQR) number of school-days lost in the households of the case-patients was 4 days (3–6); 3 days (1–6) among inpatients vs. 4 days (3–6) among outpatient (Table 1).

#### Cost of influenza-associated illness

We found differences in the distributions of the costs per-episode of influenza by site among outpatients (mean costs ranged from US$ 12.29–47.78; *p* = 0.002), but no differences among inpatients (mean costs ranged from US$ 110.82–130.97; *p* = 0.461).). We also found no statistically significant difference when we compared costs by influenza type [the median (IQR) was 91.34 (69.68–140.12) vs. 106.23 (39.57–170.05); *p* = 0.832) for influenza A and B respectively among inpatients, and 10.45 (4.60–25.45) vs. 13.67 (6.00–18.56); *p* = 0.865) for influenza A and B respectively among outpatients]. These comparisons excluded those who were co-infected. Similarly, we did not find statistically significant differences in costs by the sub-types of influenza A positive cases [the median (IQR) was 91.35 (68.13–141.30) for A(H1N1) pdm09 vs. 108.16 (75.83 - 135.88) for A(H3N2); *p* = 0.790 among inpatients, and 9.25 (4.18 - 22.34) for A(H1N1) pdm09 vs. 12.71 (6.64 - 31.92) for A(H3N2); *p* = 0.093 among outpatients].

The overall mean (SD) cost per episode among hospitalized patients was US$117.86 (88.04): < 5 years = US$114.25 (90.03); and ≥ 5 years = US$137.45 (76.24) (Table 2 and Additional file 2: Table S2). The mean (SD) cost per episode among outpatients overall was US$19.82 (27.29): < 5 years = US$21.49 (31.42); and ≥ 5 years = US$16.79 (17.30) (Table 3 and Additional file 2: Table S3). The health facility service delivery mean (SD) costs among inpatients were estimated at US$59.19 (59.39), and were thirteen times higher compared to outpatients (US$4.34 [1.30]). Overall, the mean fraction of the total cost-per-episode of influenza-associated illness relative to the average monthly household income was 60% (95% CI 45–75) among hospitalized patients. However, when the costs that were paid by the government for children < 5 years were excluded, cost-per-episode related to monthly income was 40% (95% CI 34–46). Cost-per-episode for outpatients relative to monthly income was 12% (95% CI 10–14); 11% (95% CI 9–13) if costs paid by the government for children < 5 years were excluded.

**Table 2:** Overall costs (US$^d^), including direct and indirect costs, related to influenza-associated illness among inpatients in Kenya, July 2013–August 2014

Expenditure item	< 5 years			≥5 years			Total		
	n	Mean (SD)	Range (min-max)	n	Mean (SD)	Range (min-max)	n	Mean (SD)	Range (min-max)
Direct cost	76	75.42 (71.10)	6.45–573.17	14	75.45 (36.27)	23.89–143.91	90	75.43 (66.73)	6.45–573.17
Healthcare prior to hospital/clinic visit	88	7.59 (16.95)	0.00–111.11	17	6.56 (8.48)	0.00–27.78	105	7.42 (15.86)	0.00–111.11
Total facility-based medical costs	83	59.81 (63.60)	6.45–561.50	15	55.77 (27.05)	14.67–117.90	98	59.19 (59.39)	6.45–561.50
Medications	83	13.90 (52.47)	0.33–480.23	15	18.31 (17.45)	0.33–64.78	98	14.58 (48.72)	0.33–480.23
Hospital bed fees	83	22.11 (13.95)	2.22–62.22	15	15.70 (6.13)	2.22–25.00	98	21.13 (13.24)	2.22–62.22
Procedure fees (non−/surgical)	83	2.48 (5.03)	0.00–41.11	15	2.44 (2.19)	0.00–5.56	98	2.48 (4.70)	0.00–41.11
Diagnostic tests	83	4.59 (9.72)	0.00–82.78	15	7.84 (10.33)	0.00–38.00	98	5.09 (9.83)	0.00–82.78
Routine health facility service management cost^a^	83	16.59 (10.24)	3.23–45.16	15	11.40 (4.02)	3.23–19.35	98	15.80 (9.72)	3.23–45.16
Total transportation costs	88	4.68 (8.02)	0.00–56.67	17	6.86 (9.78)	0.00–36.67	105	5.03 (8.32)	0.00–56.67
Personal car/taxi	10	10.88 (9.51)	0.56–32.22	5	23.91 (28.40)	0.67–66.67	15	15.22 (18.14)	0.56–66.67
Matatu/bus	70	9.39 (9.79)	0.89–67.78	17	6.74 (5.35)	0.67–17.89	87	8.87 (9.13)	0.67–67.78
Motorbike/bike/tuktuk	15	5.36 (5.08)	1.11–17.78	2	16.39 (12.18)	7.78–25.00	17	6.66 (6.73)	1.11–25.00
Healthcare after discharge	88	3.25 (5.95)	0.00–34.44	17	3.43 (7.53)	0.00–31.11	105	3.28 (6.19)	0.00–34.44
Child care cost	88	0.14 (0.81)	0.00–5.56	17	0.00 (0.00)	0.00–0.00	105	0.11 (0.75)	0.00–5.56
Indirect cost (Average household productivity loss^b^)	81	38.94 (38.59)	0.00–187.50	15	58.61 (53.37)	11.11–180.56	96	42.01 (41.54)	0.00–187.50
Total cost per episode^c^	76	114.25 (90.03)	9.89–670.39	14	137.45 (76.24)	36.86–287.57	90	117.86 (88.04)	9.89–670.39
Total cost per episode paid out of pocket	76	76.45 (55.13)	2.22–302.22	14	137.45 (76.24)	36.86–287.57	90	85.94 (62.49)	2.22–302.22

*SD* Standard Deviation, *NA* Not applicable; ^a^Routine healthcare facility management costs for healthcare service costs per day (equipment maintenance, electricity, water, stationary, e.t.c); ^b^Estimated by multiplying the average days when the household lost income opportunities by the household average daily income; ^c^Sum of direct and indirect costs

^d^1 US$ = 90 Kenya Shillings

**Table 3:** Overall costs (US$^d^), including direct and indirect costs, related to influenza-associated illness among outpatients in Kenya, July 2013–August 2014

Expenditure item	< 5 years			≥5 years			Total		
	n	Mean (SD)	Range (min-max)	n	Mean (SD)	Range (min-max)	n	Mean (SD)	Range (min-max)
Direct cost	100	8.62 (6.15)	3.23–33.00	55	5.97 (4.07)	3.23–23.23	155	7.68 (5.63)	3.23–33.00
Healthcare prior to hospital/clinic visit	110	2.04 (4.67)	0.00–27.78	58	0.17 (0.82)	0.00–5.56	168	1.39 (3.90)	0.00–27.78
Total facility-based medical costs	108	4.30 (1.27)	3.23–9.67	57	4.43 (1.36)	3.23–8.89	165	4.34 (1.30)	3.23–9.67
Medications	108	0.96 (1.11)	0.13–5.77	57	1.10 (1.20)	0.04–5.11	165	1.01 (1.14)	0.04–5.77
Hospital bed fees	–	NA	NA	–	NA	NA	–	NA	NA
Procedure fees (non−/surgical)	108	0.00 (0.00)	0.00–0.00	57	0.00 (0.00)	0.00–0.00	165	0.00 (0.00)	0.00–0.00
Diagnostic tests	108	0.11 (0.51)	0.00–5.00	57	0.11 (0.24)	0.00–1.11	165	0.11 (0.43)	0.00–5.00
Routine health facility service management cost^a^	108	3.23 (0.00)^e^	3.23–3.23	57	3.23 (0.00)^e^	3.23–3.23	165	3.23 (0.00)^e^	3.23–3.23
Total transportation costs	112	0.48 (0.83)	0.00–4.44	58	0.25 (0.94)	0.00–5.11	170	0.40 (0.87)	0.00–5.11
Personal car/taxi	1	NE	NE	0	NE	NE	1	NE	NE
Matatu/bus	42	3.91 (8.42)	0.44–41.67	6	8.24 (9.52)	0.22–23.33	48	4.45 (8.58)	0.22–41.67
Motorbike/bike/tuktuk	17	2.59 (1.95)	0.89–6.67	5	3.33 (1.96)	1.67–6.67	22	2.76 (1.93)	0.89–6.67
Healthcare after discharge	112	2.46 (4.77)	0.00–32.22	58	1.19 (3.48)	0.00–19.44	170	2.03 (4.40)	0.00–32.22
Child care cost	112	0.04 (0.42)	0.00–4.44	58	0.13 (0.78)	0.00–5.56	170	0.07 (0.57)	0.00–5.56
Indirect cost (Average household productivity loss^b^)	106	13.87 (31.36)	0.00–222.22	56	10.88 (16.65)	0.00–69.44	162	12.84 (27.17)	0.00–222.22
Total cost per episode^c^	100	21.49 (31.42)	3.23–235.00	55	16.79 (17.30)	3.23–72.80	155	19.82 (27.29)	3.23–235.00
Total cost per episode paid out of pocket	100	19.21 (30.70)	0.56–228.44	55	16.79 (17.30)	3.23–72.80	155	18.35 (26.69)	0.56–228.44

*SD* Standard Deviation, *NA* Not applicable, *NE* Not estimated; ^a^Routine healthcare facility management costs for healthcare service costs per day (equipment maintenance, electricity, water, stationary, e.t.c); ^b^Estimated by multiplying the average days when the household lost income opportunities by the household average daily income; ^c^Sum of direct and indirect costs

^d^1 US$ = 90 Kenya Shillings; ^e^No variability as the same amount was used for all patients

Direct mean (SD) costs associated with influenza were ten times higher for hospitalizations (US$ 75.43 [66.73]) compared to outpatient visits (US$ 7.68 [5.63]). The mean (SD) cost associated with hospitalization was US$75.42 (71.10) for children < 5 years and US$75.45 (36.27) for persons ≥5 years (Table 2 and Additional file 2: Table S2). For outpatient visits the mean (SD) cost was US$8.62 (6.15) for children < 5 years, and US$5.97 (4.07) for persons ≥5 years (Table 3 and Additional file 2: Table S3). The overall mean (SD) indirect cost-per-episode of influenza-associated illness was US$42.01 (41.54) (< 5 years = US$38.94 [38.59]; and ≥ 5 years = US$58.61 [53.37]) among hospitalized patients compared to US$12.84 (27.17) (< 5 years = US$13.87 [31.36]; and ≥ 5 years = US$10.88 [16.65]) among outpatients.

#### National economic burden of influenza-associated illness

Assuming the lowest (< 5 years = 2.7 per 1000 children; ≥5 years = 0.2 per 1000 persons) and highest (< 5 years = 4.7 per 1000 children; ≥5 years = 0.4 per 1000 persons) published incidence of hospitalizations associated with influenza activity in Kenya [15], we estimated total hospitalizations to range from 25,154 to 45,672 (< 5 years = 17,875 - 31,115; ≥5 years = 7279 - 14,557). These would result in costs ranging from US$ 2.96 to 5.37 million (< 5 years = US$2.04–3.55 million; ≥5 years = US$1.00–1.99 million) (Table 4). Similarly, assuming lowest (< 5 years = 21.8 per 1000 children; ≥5 years = 4.3 per 1000 persons) and highest rates (< 5 years = 58.0 per 1000 children; ≥5 years = 26.0 per 1000 persons) [15], we estimated that outpatient visits associated with influenza would range from 300,813 to 1,330,200 [< 5 years = 144,322 - 383,977; ≥5 years = 156,491 - 946,223]. These would translate to a total cost ranging from US$5.96 to 26.35 million (< 5 years = US$3.09–8.23 million; ≥5 years = US$2.64–15.96 million). The overall cost associated with influenza (combining in- and outpatients) could range from US$ 8.92 to 31.72 million.

**Table 4:** Estimated annual costs (in millions of 2014 US$) of influenza-associated illnesses in Kenya

Cost Item (per episode)	Hospitalized patients				Outpatients			
	Best-case scenario		Worst-case scenario		Best-case scenario		Worst-case scenario	
	Estimated cases	Cost (US$^d^ Millions) (95% CI^c^)	Estimated cases	Cost (US$^d^ Millions) (95% CI^c^)	Estimated cases	Cost (US$^d^ Millions) (95% CI^c^)	Estimated cases	Cost (US$^d^ Millions) (95% CI^c^)
Children < 5 years^a^	17,875		31,115		144,322		383,977	
Total direct costs		1.34 (1.10–1.63)		2.34 (1.92–2.84)		1.25 (1.09–1.41)		3.33 (2.89–3.76)
Facility-based medical costs		1.08 (0.89–1.34)		1.87 (1.54–2.33)		0.62 (0.59–0.66)		1.65 (1.56–1.75)
Transportation costs		0.08 (0.06–0.12)		0.15 (0.10–0.21)		0.07 (0.05–0.09)		0.18 (0.13–0.24)
Other costs^b^		0.20 (0.13–0.27)		0.34 (0.23–0.47)		0.66 (0.48–0.85)		1.76 (1.27–2.25)
Total indirect		0.70 (0.56–0.85)		1.21 (0.97–1.48)		2.01 (1.23–2.97)		5.35 (3.28–7.91)
Total costs		2.04 (1.73–2.41)		3.55 (3.01–4.19)		3.09 (2.32–4.07)		8.23 (6.18–10.84)
Persons ≥5 years^a^	7279		14,557		156,491		946,223	
Total direct costs		0.55 (0.41–0.69)		1.10 (0.81–1.37)		0.93 (0.78–1.11)		5.64 (4.72–6.69)
Facility-based medical costs		0.41 (0.32–0.53)		0.81 (0.63–1.07)		0.69 (0.64–0.75)		4.20 (3.89–4.55)
Transportation costs		0.05 (0.02–0.09)		0.10 (0.04–0.18)		0.04 (0.01–0.08)		0.24 (0.05–0.50)
Other costs^b^		0.07 (0.04–0.11)		0.15 (0.07–0.22)		0.23 (0.10–0.40)		1.42 (0.63–2.43)
Total indirect		0.43 (0.26–0.65)		0.86 (0.51–1.30)		1.69 (1.09–2.40)		10.24 (6.61–14.49)
Total costs		1.00 (0.71–1.30)		1.99 (1.42–2.59)		2.64 (1.99–3.38)		15.96 (12.02–20.43)
All ages	25,154		45,672		300,813		1,330,200	
Total direct costs		1.89 (1.60–2.25)		3.44 (2.90–4.09)		2.32 (2.08–2.58)		10.24 (9.18–11.40)
Total indirect		1.06 (0.86–1.28)		1.92 (1.55–2.33)		3.87 (2.71–5.24)		17.11 (11.98–23.17)
Total costs		2.96 (2.57–3.41)		5.37 (4.66–6.19)		5.96 (4.80–7.49)		26.35 (21.22–33.14)

^a^Estimated using published rates of influenza-associated disease in Kenya [hospitalization rate (< 5 years = 2.7–4.7 per 1000; ≥5 years = 0.2–0.4 per 1000) and outpatient rate (< 5 years = 13.0–58.0 per 1000; ≥5 years = 4.3–26.0 per 1000)]; ^b^Combines costs for childcare, and healthcare seeking prior and after hospitalization or outpatient visit; ^c^Mean cost and 95% confidence intervals estimated using 1000 bootstrap samples

^d^1 US$ = 90 Kenya Shillings (2014)

Best-case scenario: Assuming the lowest published incidence rate of influenza-associated illness

Worst-case scenario: Assuming the highest published incidence rate of influenza-associated illness

## Discussion

This is the first estimate of the economic impact associated with medically-attended influenza in Kenya. We found that, depending on annual influenza virus circulation, the costs associated with influenza in Kenya could be as high as US$ 32 million. We estimated that the overall mean cost per episode was US$118 for hospitalization and US$20 for outpatient visits, a substantial burden for Kenyan families when we consider their average monthly income, the loss of self-employment wages by missed days at work, and that most of medical costs are paid out-of-pocket. Influenza vaccine is the most effective way to prevent influenza and should be considered for groups at risk of influenza-associated complications and hospitalizations in Kenya.

The overall cost-per-episode of influenza-associated hospitalizations was six times higher when compared to outpatient visits. This was driven by the facility-based medical cost component, where the hospitalization cost was thirteen-fold higher, and was similar to the results published in Bangladesh [13]. Because of the higher frequency of outpatient visits, the annual economic burden for outpatient influenza-associated illness in Kenya was substantially high relative to hospitalizations, which is consistent with results reported from other studies [3, 13]. However, the overall cost-per-episode of influenza in our study was lower than reported elsewhere in developed countries [5, 18]. This could be explained by the relative lower cost of healthcare and the comparatively low income level in Kenya where the gross national income per capita is estimated at US$939 [19].

The duration of hospitalization was higher for children < 5 years compared to older persons. This is contrary to findings reported elsewhere [6] and may be explained by the fact that in our study population only 7 persons were > 40 years old. Older adults tend to stay longer in hospital due to complications associated with underlying diseases [20]. We also found that the cost-per-episode when the illness involved a child < 5 years old was similar to the cost among older patients aged ≥5 years. Other than the effect of underrepresentation of older patients in our study, this finding could also be explained by the fact that a higher percentage (46%) of children < 5 years compared to older patients (20%) had sought healthcare or had drugs bought over the counter prior to the hospitalization or outpatient visit. The fact that an influenza-associated illness involving young children – who are at a high risk of influenza-associated complications [21, 22] – also results in high economic cost highlights the need for the development of targeted vaccination and other preventive strategies among this age group.

Influenza-associated illness also resulted in school absenteeism among sick children, with a median of 4 days of missed school, which was comparable to findings from a study conducted in Hong Kong [23], and another study conducted in the US [4]. Notably, we found that school absenteeism was higher among households of outpatients compared to those of hospitalized patients. A possible explanation could be that older siblings may be asked to stay at home and take care of the younger ones as the caretakers take the sick child to a health facility. However, in a case of hospitalization - where it may not be certain when the mother/parent will return - young children may be left at the care of relatives or neighbors.

Overall, a single episode of influenza-associated hospitalization resulted in a substantial cost of approximately 60% of the household average monthly income while outpatient-associated influenza costs represented 12%. Regardless of the fact that medical costs at the hospital were covered by the government for children < 5 years [12], the overall resultant costs from an influenza-associated hospitalization – which were paid out-of-pocket (40% of the household average monthly income) – could put a financial strain on families [24] and may also negatively impact on other competing household priorities such as food and education. Our data showed that approximately 64% of household members lost days at work which in many cases imply lost income as most were self-employed. Considering the possibilities of influenza-associated complications, the financial impact to the household arising from such cases could even be greater.

The mean cost-per-episode of influenza among children < 5 years in our study (US$114) was similar to the cost reported in a study of malaria patients that was conducted in Kenya in 2009 which reported a mean cost of US$100 [25]. However, there were some differences in the methodologies of these studies that would limit our ability to make direct comparisons. Unlike in our study, death was included in the household indirect cost where it was calculated as the net present value of future potential earnings. The resultant total indirect cost accounted for 60% of the overall mean cost per episode of malaria.

Our study had some important limitations. Our study, incorporated data collected only from one year, and to account for potential variation on influenza circulation from year to year, we used a sensitivity analysis based on the range of previously estimated rates of influenza hospitalization in Kenya. Moreover, due to the small sample size, we were not able to breakdown costs associated with influenza by small age groups. The sample size may also have affected our ability to find any differences in influenza-associated costs by type and sub-type of viruses; some studies have suggested differences in severity of influenza-associated disease based on type and subtype of virus which could lead to differences in costs [26, 27]. Another potential limitation was that older patients were underrepresented in our patient-population as healthcare seeking is low in this group [28], and our cost estimates associated with influenza could be underestimated, principally considering the high prevalence of underlying medical conditions among older patients that could lead to prolonged hospitalization. Indeed, as routinely seen in our hospital-based surveillance, only 3% of the study participants were aged ≥40 years and only 5% of the study participants had been tested for HIV. Additionally, data on other underlying comorbidities such as asthma, diabetes, cardiac disease, and tuberculosis were limited. With regard to the calculation of costs, we estimated the facility-based medical-costs by applying charges that were recorded on the hospital bill charge sheets, the price catalogue charts, and the receipts that were issued to the patients as a proxy for the actual cost. As such, the actual costs may have varied by the extent to which these charges approximated the true cost to the hospital. Moreover, we did not include the physician’s fees in our analysis. We also did not incorporate the indirect cost of days with reduced activity among case-patients, productivity loss for non-income generating activities, and deaths in our data collection and subsequently in the analysis. This likely further served to underestimate the actual costs associated with influenza. The self-reported costs on prior expenditures and post-discharge expenditures could have suffered some degree of under- or over reporting as they were not substantiated by receipts. Lastly, we did not include costs incurred by persons who did not seek care because of influenza illness; costs of self-medication within the community and related loss of productivity by these persons went unmeasured.

## Conclusions

Our findings show that medically attended, influenza-associated illness in Kenya generate substantial direct and indirect costs. The burden is driven mostly by outpatient visits. Whereas this study highlights an important societal economic impact of influenza-associated illness, further studies should explore the cost-effectiveness of targeted influenza vaccination in Kenya and account for years lost due to death or disability in order to guide vaccine recommendation policies.

## Additional files

Additional file 1: Supplemental methods. (DOCX 28 kb)

Additional file 2: Supplemental tables. (DOCX 26 kb)

## Abbreviations

BD: Becton Dickson
CDC: U.S. Centers for Disease Control and Prevention
CI: Confidence interval
CRH: County Referral Hospital
ILI: Influenza-like illness
IQR: Interquartile range
IRB: Institutional review board
KEMRI: Kenya Medical Research Institute
RDT: Rapid diagnostic test
rtRT-PCR: Real-time reverse-transcription polymerase chain reaction
SARI: Severe acute respiratory illness
SD: Standard deviation
US$: USA dollar
WHO: World Health Organization
