# Supplementary material for: The cost of influenza-associated hospitalizations and outpatient visits in Kenya
Source: BMC Public Health. 2019 May 10;19(Suppl 3):471. doi: 10.1186/s12889-019-6773-6 (PMC6696702; doi:10.1186/s12889-019-6773-6)
Supplement: Supplementary file 1 — Supplemental methods. (DOCX 28 kb) [file 12889_2019_6773_MOESM1_ESM.docx]

**Appendix S1. Supplemental Methods**

**Estimation of the routine service delivery cost**

The routine service delivery cost – which included buildings and equipment maintenance, transport, electricity, water, fuel, communication, stationery, and wages for support staff – was estimated per day for each patient using the following steps:

1. Using a structured questionnaire, we obtained data on the actual service delivery costs covering the items mentioned above from the administrative personnel at the five study sites for the financial year 2014 (FY2014).
2. From the health records office, we obtained data on the total bed occupancy (daily sum of the patients who were hospitalized) and the outpatient visits over the same period (FY2014).
3. We then calculated the average cost per patient by dividing the total annual cost (combining data from all the five sites) by the sum total of hospitalized patients and outpatients seen each day at the five facilities over the period FY2014.
4. For hospitalized patients in our study, we then multiplied the estimate calculated in step 3 above by the number of days the patients was hospitalized. For outpatients, the estimate calculated in step 3 was used as the cost of routine health facility service delivery.

To minimize the potential for double counting costs, registration fees were not included in the analysis of the facility-based medical fees as such

**Estimation of the lost opportunity cost**

To estimate the household lost opportunity cost, we used data on average monthly household income and number of people who missed work or income opportunities due to the illness of the case-patient. Studies conducted elsewhere have limited their calculation of opportunity loses to the case-patient and/or their care taker [[1-3](#_ENREF_1)]. In our context, we thought that this would potentially underestimate the cost involved as household members tend to play an important role in an illness episode which may include absenteeism from work and school [[4](#_ENREF_4)]. We used the following steps to calculate the household opportunity cost:

1. For each household of the case-patient enrolled in our study, we collected data on the average monthly household income. We also collected data for each person in the household (including the case-patient) who missed work or income opportunities as a result of the illness of the case-patient, and the total number of days when they missed such opportunities.
2. As the next step, we calculated the household average number of days of missed work opportunities by dividing the sum total of the number of days of work missed by all the household members by the number of people in the household who reported to be engaged in an income generating activity.
3. We then calculated the average daily household income by dividing the average monthly household income by 30 days.
4. The average daily household income was then multiplied by the average number of days of missed work opportunities in the household as calculated in step 2 above to estimate overall opportunity cost over the duration of the illness episode.

We also considered using data on the individual-based opportunity cost reported for each of the household members who missed work opportunities but refrained from using the data as we found that some of the numbers reported were unusually high and in contradiction to the reported monthly housed income.

**Comparison of cost data estimated with and without WHO-CHOICE estimates**

We explored the option of using the WHO-CHOICE estimates for health facility service delivery costs [[5](#_ENREF_5)]. However, we found minimal differences when we used actual data collected from the study sites to estimate the health facility service delivery costs compared to when we used the WHO-Choice estimates. Among inpatients the overall mean (SD) cost per episode was US$117.86 (88.04) using the actual data compared to US$112.06 (87.41) when we used the WHO-Choice estimates (p=0.658). Among outpatients the overall mean (SD) cost per episode was US$19.82 (27.29) using the actual data compared to US$19.14 (27.31) when we used the WHO-Choice estimates (p=0.8254). Similarly, no statistically significant differences were found when we compared the overall medical costs among inpatients using these two different methods [mean (SD) = US$59.35 (61.12) using actual data compared to mean (SD) = US$53.55 (60.87) using the WHO-Choice estimates]. However, the medical costs were significantly higher among outpatients when we used the actual data [mean (SD) = US$ 4.29 (1.24)] compared to [mean (SD), US$3.60 (1.23)] when we used the WHO-Choice estimates (p<0.001).

**References**

1. Simmerman JM, Lertiendumrong J, Dowell SF, Uyeki T, Olsen SJ, Chittaganpitch M, et al. The cost of influenza in Thailand. Vaccine. 2006;24(20):4417-26. doi: 10.1016/j.vaccine.2005.12.060. PubMed PMID: 16621187.

2. Ehlken B, Anastassopoulou A, Hain J, Schroder C, Wahle K. Cost for physician-diagnosed influenza and influenza-like illnesses on primary care level in Germany--results of a database analysis from May 2010 to April 2012. BMC public health. 2015;15:578. doi: 10.1186/s12889-015-1885-0. PubMed PMID: 26093501; PubMed Central PMCID: PMC4475612.

3. Bhuiyan MU, Luby SP, Alamgir NI, Homaira N, Mamun AA, Khan JA, et al. Economic burden of influenza-associated hospitalizations and outpatient visits in Bangladesh during 2010. Influenza and other respiratory viruses. 2014;8(4):406-13. doi: 10.1111/irv.12254. PubMed PMID: 24750586; PubMed Central PMCID: PMC4181799.

4. World Health Organization (WHO). Who Guide to Identifying The Economic Consequences of Disease and Injury. Available at: <http://www.who.int/choice/publications/d_economic_impact_guide.pdf>. Accessed on October 4th, 2015. 2009.

5. World Health Organization (WHO). CHOosing Interventions that are Cost Effective (WHO-CHOICE): Country-specific unit costs. Available at: <http://www.who.int/choice/country/country_specific/en/>. Accessed on Aug 14th, 2015 2011.
