# Supplementary material for: The cost of influenza-associated hospitalizations and outpatient visits in Kenya
Source: BMC Public Health. 2019 May 10;19(Suppl 3):471. doi: 10.1186/s12889-019-6773-6 (PMC6696702; doi:10.1186/s12889-019-6773-6)
Supplement: Supplementary file 2 — Supplemental tables. (DOCX 26 kb) [file 12889_2019_6773_MOESM2_ESM.docx]

**Supplemental** **Table 1:** Definition and data source of key study variables

| **Variable** | **Definition** | **Rationale** | **Data source** |
| --- | --- | --- | --- |
| Facility-based medical costs | All medical costs incurred at the facility. Includes registration fees, cost of medications, hospital bed fees, medical procedure costs, diagnostics, and Healthcare service delivery cost. Costs were calculated up to a maximum of 14 days from the date when the patient was tested for influenza. Costs among children<5 years are paid for by the government while costs among persons >5 years are borne by the patient. There were no extra charges on hospital bed fees for caretakers inpatients | Component of direct costs | These data were collected at the time of discharge by referring to the patient files, hospital discharge billing sheets and outpatient receipts, catalogue of prices for medications and laboratory tests. Additional costs of medication collected during follow-up interviews for patients who sought healthcare after discharge. |
| Routine service delivery costs | Cost of routine management of the health facility including (buildings and equipment management, transport, electricity, water, fuel, communication, Stationery, wages for support staff). These costs are borne by the government | Component of direct costs | Health facility administrative records |
| Healthcare cost prior to hospital/clinic visit | Total costs (medications and transportation) associated with current illness episode for which healthcare was sought prior to hospitalization or outpatient visit. These costs are borne by the patient | Component of direct costs | Self-reported in the initial interview upon enrolment |
| Post-discharge healthcare cost | Total costs (medications and transportation) to the household associated with current illness episode for which healthcare was sought after the case-patient was discharged and to a maximum of 14 days from the date of testing for influenza. These costs are borne by the patient | Component of direct costs | Self-reported costs during follow-up interviews. Excludes medication costs for the days the patient was still hospitalized |
| Transport cost | Costs to facilitate movement to and from the medical facility or place of seeking care by the patient and household members. Includes both private and public means. These costs are borne by the patient | Component of direct costs | self-reported during the initial interview and follow-up interviews |
| Child care cost | Cost paid for childcare of other children in the household while the respondent was ill or taking care of the patient. These costs are borne by the patient | Component of direct cost | Self-reported during the initial interview and follow-up interviews |
| Lost work days | Workdays lost by any of the household members due to influenza-associated illness | Component of in-direct costs and used to estimate the household productivity loss cost | Self-reported during the initial interview and follow-up interviews |
| Household lost work days | Average number of days when the household missed work opportunities | Used to estimate the household productivity loss cost | Collected during the initial and follow-up interviews. Calculated as the total numbers of work days opportunities missed by all the household members divided by the number of people in the household who lost such opportunities |
| Household daily income | Self-reported daily income for the household | Used to estimate the household productivity loss cost. The midpoint value of the income category was used to represent household income | Estimated from the self-reported household monthly income. For each case-patient, the daily income was calculated by dividing the average monthly income amount (for each income category reported) by 30 days. The income categories were ≤5,000; 5,001-10,000; 10,001-25,000; 25,001-50,000; 50,001-100,000 and ≥100,000. |
| Household productivity loss | The monetary loss of productivity per household due the illness | Component of indirect costs | Self-reported during the initial interview and follow-up interviews |
| Lost school days | School days not attended by any member of the household due to influenza infection | Component of indirect costs but was not quantified in monetary terms | Self-reported during the initial interview and follow-up interviews |

**Supplemental** **Table 2**: Overall costs, direct and indirect costs, related to influenza-associated illness among inpatients, Jul 2013 - Aug 2014

|  | Costs among inpatients (US$^ⱡ^ ) | | | | | |
| --- | --- | --- | --- | --- | --- | --- |
| Expenditure item | <5 years | | ≥5 years | | Total | |
|  | **n** | Median(IQR) | **n** | Median(IQR) | **n** | Median(IQR) |
| Direct cost | 76 | 64.96(38.18-94.80) | 14 | 78.85(45.23-88.57) | 90 | 65.31(39.57-90.47) |
| Healthcare prior to hospital/clinic visit | 88 | 2.11(0.00-7.78) | 17 | 5.56(0.00-7.22) | 105 | 2.11(0.00-7.78) |
| Total facility-based medical costs | 83 | 52.12(28.90-73.24) | 15 | 54.68(38.57-60.12) | 98 | 52.46(32.79-69.79) |
| Medications | 83 | 3.89(1.11-15.11) | 15 | 17.56(2.44-22.00) | 98 | 5.95(1.11-16.78) |
| Hospital bed fees | 83 | 20.00(11.11-30.00) | 15 | 15.00(11.11-20.00) | 98 | 20.00(11.11-27.78) |
| Procedure fees (non-/surgical) | 83 | 0.56(0.00-4.44) | 15 | 3.33(0.00-4.44) | 98 | 0.56(0.00-4.44) |
| Diagnostic tests | 83 | 2.22(0.00-4.44) | 15 | 5.56(0.00-15.56) | 98 | 2.22(0.00-5.00) |
| Routine health facility service management  cost^a^ | 83 | 16.13(9.68-22.58) | 15 | 9.68(9.68-12.90) | 98 | 12.90(9.68-19.35) |
| Total transportation costs | 88 | 2.22(0.00-6.39) | 17 | 2.22(0.78-7.78) | 105 | 2.22(0.00-7.00) |
| Personal car/taxi | 10 | 8.00(5.56-11.11) | 5 | 11.11(2.22-38.89) | 15 | 10.00(4.44-22.22) |
| Matatu/bus | 70 | 6.67(3.33-13.33) | 17 | 4.67(2.33-12.00) | 87 | 6.67(3.00-12.67) |
| Motorbike/bike/tuktuk | 15 | 3.33(2.22-6.11) | 2 | 16.39(7.78-25.00) | 17 | 3.33(2.67-7.78) |
| Healthcare after discharge | 88 | 0.50(0.00-3.89) | 17 | 0.00(0.00-3.89) | 105 | 0.33(0.00-3.89) |
| Child care cost | 88 | 0.00(0.00-0.00) | 17 | 0.00(0.00-0.00) | 105 | 0.00(0.00-0.00) |
| Indirect cost (Average household productivity loss^b^) | 81 | 25.93(11.11-58.33) | 15 | 38.89(19.44-77.78) | 96 | 30.09(12.04-59.72) |
| Total cost per episode^c^ | 76 | 91.35(69.07-136.54) | 14 | 122.39(75.01-163.35) | 90 | 92.98(69.45-141.30) |
| Total cost per episode paid out of pocket | 76 | 60.85(39.33-88.41) | 14 | 122.39(75.01-163.35) | 90 | 68.00(45.93-107.22) |

IQR – Interquartile Range; NA-Not applicable; ^a^Routine healthcare facility management costs for healthcare service costs per day (equipment maintenance, electricity, water, stationary, e.t.c); ^b^Estimated by multiplying the average days when the household lost income opportunities by the household average daily income; ^c^Sum of direct and indirect costs.

^ⱡ^ 1 US$=90 Kenya Shilings

**Supplemental** **Table 3**: Overall costs, direct and indirect costs, related to influenza-associated illness among outpatients, Jul 2013 - Aug 2014

|  | | Costs (US$^ⱡ^ ) | | | | | |
| --- | --- | --- | --- | --- | --- | --- | --- |
| Expenditure item | | <5 years | | ≥5 years | | Total | |
|  | | **n** | Median(IQR) | **n** | Median(IQR) | **n** | Median(IQR) |
| Direct cost | | 100 | 6.39(4.14-11.11) | 55 | 3.95(3.67-6.95) | 155 | 5.47(3.73-10.17) |
| Healthcare prior to hospital/clinic visit | | 110 | 0.00(0.00-1.89) | 58 | 0.00(0.00-0.00) | 168 | 0.00(0.00-0.00) |
| Total facility-based medical costs | | 108 | 3.73(3.50-4.64) | 57 | 3.83(3.50-5.56) | 165 | 3.80(3.50-4.75) |
| Medications | | 108 | 0.50(0.28-1.38) | 57 | 0.60(0.28-1.78) | 165 | 0.58(0.28-1.44) |
| Hospital bed fees | | - | N/A | - | N/A | - | N/A |
| Procedure fees (non-/surgical) | | 108 | 0.00(0.00-0.00) | 57 | 0.00(0.00-0.00) | 165 | 0.00(0.00-0.00) |
| Diagnostic tests | | 108 | 0.00(0.00-0.00) | 57 | 0.00(0.00-0.00) | 165 | 0.00(0.00-0.00) |
| Routine health facility service management  cost^a^ | | 108 | 3.23(3.23-3.23)* | 57 | 3.23(3.23-3.23)* | 165 | 3.23(3.23-3.23)* |
| Total transportation costs | | 112 | 0.00(0.00-0.78) | 58 | 0.00(0.00-0.00) | 170 | 0.00(0.00-0.56) |
| Personal car/taxi | | 1 | 0.56(0.56-0.56) | 0 | - | 1 | 0.56(0.56-0.56) |
| Matatu/bus | | 42 | 1.72(1.11-2.78) | 6 | 5.00(0.56-15.33) | 48 | 1.72(1.11-3.22) |
| Motorbike/bike/tuktuk | | 17 | 1.56(1.11-4.44) | 5 | 2.78(2.22-3.33) | 22 | 1.94(1.11-4.44) |
| Healthcare after discharge | | 112 | 0.00(0.00-4.17) | 58 | 0.00(0.00-0.00) | 170 | 0.00(0.00-1.89) |
| Child care cost | | 112 | 0.00(0.00-0.00) | 58 | 0.00(0.00-0.00) | 170 | 0.00(0.00-0.00) |
| Indirect cost (Average household productivity loss^b^) | | 106 | 0.00(0.00-16.67) | 56 | 2.78(0.00-13.89) | 162 | 0.00(0.00-16.67) |
| Total cost per episode^c^ | | 100 | 11.36(4.94-24.61) | 55 | 9.23(4.60-23.51) | 155 | 10.89(4.75-23.78) |
| Total cost per episode paid out of pocket | | 100 | 8.056(4.19-22.78) | 55 | 9.23(4.60-23.51) | 155 | 8.67(4.18-23.33) |
| IQR – Interquartile Range; NA-Not applicable; ^a^Routine healthcare facility management costs for healthcare service costs per day (equipment maintenance, electricity, water, stationary, e.t.c); ^b^Estimated by multiplying the average days when the household lost income opportunities by the household average daily income; ^c^Sum of direct and indirect costs. | | | | | | | |
| ^ⱡ^ 1 US$=90 Kenya Shilings; *No variability as the same amount was used for all patients | | | | | | | |
